# Clitoral reconstruction: challenges and new directions

**DOI:** 10.1038/s41443-022-00572-6

**Published:** 2022-04-13

**Authors:** Michela Villani

**Affiliations:** HES-SO, School of Social Work Fribourg, Delémont, Switzerland

**Keywords:** Therapeutics, Quality of life

## Abstract

Clitoral reconstruction (CR) has been the subject of several studies in recent years, mainly in the medical field. Women with female genital mutilation or cutting (FGM/C) seek clitoral reconstructive surgery to improve their sexual well-being, but also because they are affected by poor self- and body image. CR is supposed to help women with FGM/C reconstruct their sense of self, but the benefits and risks of this surgery have not been sufficiently explored. There are currently no recommendations supporting CR from mainstream medical bodies, and there have been very few ethical studies of the procedure. This article critically discusses the principal studies produced in the medical field and available reflections produced in the social sciences. Through the theoretical frameworks of postcolonial and feminist studies, the article discusses sexuality and pleasure, gender and identity, and race and positionality, with the aim of promoting collaborative work on CR between researchers and social and health professionals.

Healthcare services for women with a female genital mutilation/cutting (FGM/C) have proliferated across Europe in recent decades [1–3]. In terms of case management, two surgical techniques are practiced: (1) defibulation [4] and (2) clitoral reconstructive surgery [5]. Depending on the type of FGM/C (WHO has classified four major types of FGM/C: Type 1 refers to partial or total removal of the clitoral glans and/or the prepuce/clitoral hood; Type 2 (Excision): partial or total removal of the clitoral glans and labia minora, with or without excision of labia majora; Type 3 (Infibulation): narrowing of the vaginal opening by cutting and repositioning the labia minora and/or labia majora to create a covering seal, with or without removal of the clitoral glans and/or the prepuce/clitoral hood; Type 4: all other forms of harmful procedures on the female genitalia. Available at https://www.who.int/news-room/fact-sheets/detail/female-genital-mutilation (accessed 17 December 2021), these surgeries are respectively supposed to (1) restore the vaginal opening to its pre-modified form and facilitate menstrual bleeding and/or childbirth, and (2) restore the function and/or aesthetics of the “uncut” clitoris, sometimes also with the aim of helping affected women feel more “whole” or “feminine” in terms of qualitative self-appraisals (albeit typically assessed non-systematically and without validated measures, such as psychometrically adequate questionnaires). Other services aim to meet the specific needs of women with FGM/C in Europe by offering psycho-sexual consultations [6, 7].

Clitoral reconstructive surgery is often managed in a multidisciplinary manner [8–11], which can turn this relatively simple procedure (i.e., from a technical or surgical perspective) into a complex repair journey [12]. While there are several terms in use [13], that of clitoral reconstruction (CR) is used today to indicate the surgery performed within a global case management framework [14] and this will be the term employed in the present essay. Although numerous studies have sought to investigate the long- and short-term physical (e.g., obstetric) repercussions of FGM/C [15] as well as potential psycho-sexual consequences [16], few studies have demonstrated whether CR is reliably effective with respect to any of its purported aims [17–20], and its risks and benefits have not been sufficiently explored [21, 22]. Currently, CR is not officially recommended by any mainstream medical or professional body [21, 23]. Additionally, or perhaps as a consequence, there is considerable unevenness in social welfare cover for the procedure depending on the country [24].

What does CR involve? The surgical technique has been modified since the original process was described [25, 26], but it essentially involves cutting into the genital area to bring forward and externalise, at least to some extent, the subcutaneous clitoris (i.e., parts of the clitoris that remain intact after FGM/C). Currently, there are 5 types of clitoral reconstruction techniques performed by multiple specialists (gynaecologists, urologists, and plastic surgeons), with little interdisciplinary communication between these different areas of expertise [21, 22]. That there has been little to no ethical consideration of CR has also been noted [21]. At present, post-operative pain management is considered to be inadequate [27]. Furthermore, during consultations, specialists have noted the existence of common misconceptions, such as mistaken ideas regarding anatomy (e.g., that the entire clitoris is absent prior to surgery, when in fact most of the organ is subcutaneous and remains intact) and a lack of basic sexual education [27, 28]. Nonetheless, requests for CR are growing, and the surgery is carried out in several European countries, in the US and throughout Africa. Given all these elements, it is not surprising that CR has been characterised as a “controversial surgical procedure” [27].

Motivations for CR are multifarious. Women with FGM/C living in the Global North sometimes experience very aggressive anti-FGM/C discourses [29, 30], which, in the context of migration, can generate a change in perception regarding FGM/C or its bodily consequences [31]. For example, women with FGM/C who, in their home countries, may have regarded the procedure as a normal, natural, or inevitable part of life, or as one that dignifies women, affirms their cultural identities, and beautifies or enhances the vulva, may later come to see the very same practice as one that is oppressive, mutilating, and sexually harmful, given how it is characteristically framed in Global North countries. This change in perspective is often accompanied by a perceived or experienced sense of stigma [32, 33], with a consequent negative impact on sexuality and sexual pleasure [34, 35]. The reasons cited by women who seek to undergo CR as a form of redress for FGM/C include poor self-image, poor body image [22], as well as questions linked to gender identity (e.g., a sense of being a ‘true woman’ or ‘fully feminine’) [36, 37]. A wish to improve sexuality in terms of desire and of sensation has also been reported [22, 38, 39], as has the wish to reduce painful sensations or to avoid embarrassment experienced during sexual relations [36].

This article seeks to establish an interdisciplinary dialogue between the principal studies produced in the medical field and the reflections produced in the social sciences. To address notions such as identity, stigma, and origins/motivations, I will call upon the theoretical tools of postcolonial studies [40]. To critically discuss questions relating to gender, the body, and transformative technologies in relation to the notion of free choice, I will draw on current debates in feminist studies. Finally, so as to approach sexuality in a comprehensive and less genitalia-focused manner, I will adopt theoretical elements of sexualities studies, which aims to position sexual phenomena within a broader psychosocial, cultural, and historical backdrop. The aim of this article is to promote both critical discussion and interdisciplinary exchange, in the hope of outlining future pathways to explore with the help of the previously mentioned tools. Its final objective is to chart possible healthcare and well-being interventions which could soon be developed, and to foster collaboration between researchers and health and social work professionals.

## Unregulated medical market and neoliberal feminism: questioning the “free choice”

The uncertainty surrounding CR, the differences in surgical techniques [21, 22] and the disparities in terms of services offered [24], pain management [27], and social welfare cover have pushed some women to seek help from private clinics or from questionable lobby groups [13, 41]. At the same time, the deregulation of sexual surgeries in the medical market - among which I include surgical reduction of the labia minora (labioplasty), perineal tightening (perineorrhaphy) and hymen reconstruction (hymenoplasty) - and the sometimes arbitrary distinction between cosmetic and reconstructive surgeries, contributes to the ambiguous status of CR. A recurring theme in the literature is the vagueness or inconsistency with which certain types of interventions come to be considered “medically necessary,” whereas others are merely “cosmetic” or otherwise elective [42]. Studies that have examined the reasons given by women for these types of surgery are similar to those given by women who have undergone FGM/C who undertake CR: the appearance of their genitalia, the embarrassment of undressing, how others consider them and unsatisfactory sexual relations [43, 44]. According to one view, the importance of women’s needs (with or without FGM/C), along with their right to fully enjoy sexual well-being according to their own values (e.g., regarding different kinds of sexed embodiment), suggests that they should be free to undertake genital modifications they regard as beneficial, as long as they give informed consent [45]. Another view holds that it is equally important to take into consideration and critically evaluate underlying norms that may pressure women into undergoing risky genital surgeries for dubious reasons (e.g., reasons reflecting gender injustice in the wider society, such as overly restrictive beauty norms that are asymmetrically applied to women) [46]. Along with this, seeming contradictions within public health policies, according to which CR and so-called ‘cosmetic’ surgeries are regarded as being categorically different from one another, in terms of motivations or outcomes, must also be thoroughly explored [47].

With regard to the first view, one feminist approach – so-called post-feminism, also seen as a “sensibility” [48] – is characterised by its marked focus on questions of empowerment and entrepreneurialism, of choice and individualism, of make-over and self-reinvention/transformation. The overlap between post-feminist thought and neoliberal ideas has been critiqued on several occasions [49, 50]. One critique holds that the interpretation of “choice” in both cases is reductively understood in terms of the personal project (i.e., of self-development or actualisation), which thus becomes integrated into logic of business: one must purchase goods and services, such as body-changing surgeries, to pursue this project, which is never fully finished or realised. According to this view, “individualising technologies,” whether clitoral reconstructive surgery or purportedly cosmetic surgeries such as elective labioplasty, are part of the range of self-transformative options that women can invoke to become more resilient in society, or even to perform better sexually. However, this critique maintains that the focus on the individual and self-transformation inappropriately downplays or makes invisible problematic social forces, such as unjust gendered expectations surrounding sexual desirability, that ought to be addressed head-on.

As discussed above, for many women seeking CR, the reconstructed clitoris is often associated with a reconstruction of the self [51, 52]. Some women who undertake CR consider it transformative: they are no longer “the same” as they were before the procedure; they are living “a new life” [52]. The surgery, along with psychosexual therapy, is alleged in some cases to bring profound change to their relationships, sexuality and even to their sense of womanhood [37]. In the Global North, contemporary psychological coaching techniques are widespread and are supposed to regulate different areas of human life. If, on the one hand, psycho-sexual counselling during CR allows women to acquire tools and techniques to transform themselves [52], attention should also be paid to the neoliberal ideology of self-made subjects deployed in multiple ways, not only through surgery. For example, we can ask whether it is desirable to live in a world in which we do not dare to be imperfect, incomplete, or to sometimes fail.

Alternatively, or additionally, perhaps there needs to be a reconsideration of which types of procedures should be classified as “mutilations,” with special attention paid to the pattern by which genital-modifying practices affecting women of the Global South tend to be described as mutilations, whereas anatomically similar practices pursued by “Western” women are regarded as being simply “cosmetic” [53]. Consider an example to highlight one such tension. A study conducted between 2014 and 2020 on 702 patients, recently published by a German surgeon, highlights a sharp increase in iatrogenic deformities after initial labia reductions [54]. This can be compounded by mistakes made during the procedure, which cause irreparable damage or problems that are difficult to correct. The main responsibility lies with practitioners, whose operations could fairly be described as resulting in “genital mutilation,” requiring substantial repairs. The study reports that 98% of women who were upset with the outcome of their labiaplasties (and asked for repair) stated that they “felt psychologically impaired by the unsightly appearance of their labia, albeit to varying degrees” [54:2457]. However, as functional impairments were evident in 77% of cases, the author concludes that “the harsh reality, that the number of patients experiencing poor outcomes and mutilations is on the rise, requires us to urgently change our approach […] The reconstruction of mutilations, such as fully amputated labia minora, is not easy and frequently produces only mediocre results. The prevention of errors during the initial operation must become a top priority as a result” [54:2462].

Another example of a case that resists easy classification is that of young Cambodian women requesting perineorrhaphy to tighten their vagina, purportedly to prevent their husband from resorting to prostitution in search of sexual satisfaction [55]. By tightening their vagina, these women hope to increase their husband’s sexual pleasure, although there are significant risks and side effects often associated with this procedure, such as permanent pain and sensitivity during intercourse. By modifying their genitals, women conform to social expectations, which should be further questioned in terms of unequal hierarchy of sexual pleasures, as well in terms of individual agency in defining the status of their own genitals (e.g., as “mutilated” or “enhanced”) and in terms of actual engagement in sexual activity (e.g., having or lacking confidence in one’s body during a sexual encounter, the possibility of negotiating sexual practices with partners, and so on).

Medical research should further explore the dimensions of well-being gained (or possibly impaired) through such genital modification practices, considered against the backdrop of morally questionable social norms [56], or even mistaken beliefs, that may drive women to seek them out [35]. Another recent study focused on the elaboration of a kit of 3D pelvic models, 2-dimensional figures of female and male sexual anatomy, and files for 3D printing that can be used in anatomy and sex education among and by health professionals, teachers, sex educators, students, and the general population. The authors acknowledge that myths, misconceptions, and taboos about sexual anatomy and physiology are common and can affect sexual health and sustain harmful practices and beliefs [28]. In response to this, some have recommend educating women—for example, women with FGM/C seeking CR to “restore” what they take to be a missing clitoris—about their genital anatomy and “disabusing” them of the myths surrounding female sexual functions [21].

While it may be urgent to provide adequate education to women in matters of sexuality, little emphasis is placed on ignorance within the medical profession [57], or even on obstetrical-gynaecological violence [58, 59]. Gender-biased structures within Western medicine have also impeded knowledge of clitoral anatomy for centuries, propagating racist and misogynous theories of female genital function [60, 61], and even having practiced type 2 “FGM” (clitoridectomy) for many decades in several European and North American countries on women accused of “licentious” practices such as masturbation [62]. Knowledge concerning the clitoris has been fragmentary, incomplete and even erroneous [63], which has left women and girls in many societies to grow up in ignorance of their own bodies [64].

Thus, again, it is not only the sexual or anatomical “ignorance” of women seeking CR and other genital procedures that needs to be addressed. Inadequately informed and conflicting opinions on the part of medical practitioners is also a concern. For example, a recent study of 8 gynaecologists in Sweden showed great variety in how the gynaecologists positioned themselves toward CR, with some being uncertain as to the claimed or intended benefits of the surgery despite practicing it themselves [65]. While this study undoubtedly shows the urgency of defining a standardised protocol concerning patient selection criteria and clear recommendations regarding CR, it also demonstrates that for the moment women with FGM/C in Sweden are entirely at the discretion of the gynaecologist, his or her beliefs, opinions and representations of excision and reconstruction surgery. Ultimately, they are heavily influenced his or her recommendation, which reinforces the asymmetrical power relationships between them. While women should have a right to choose to undergo cosmetic or reconstructive surgery in pursuit of their values, without undue pressure and under conditions of informed consent, it is also essential to promote a culture that respects differences with regard bodies and gender, and a thorough knowledge of sexual matters. Inadequately informed or misinformed choices cannot be considered sufficiently ‘free’.

## Who speaks in the name of whom? Displaced subjects and hybrid identities

There are many studies which cite the central role of gender, identity and a feeling of stigma perceived or experienced by women with FGM/C, which can result in them seeking CR [31, 35, 37, 38, 65]. This reason/justification is more often given by young women who were born (or arrived when very young) in Northern countries [65–68]. Clitoral reconstruction has been widely interpreted as a social practice rooted in a desire for equality [38, 39], articulated by Black women of sub-Saharan descent living in the Global North. Women living with FGM/C in European countries claim that they attribute deep symbolic value to the path of clitoral reconstruction, which is also expressed in terms of a desire to feel “complete” and to feel like “all other women.” However, this appeal to “other women” may amount to an identification with white women, whose bodies are held up as models [69]. This raises the question of racial hierarchy, replaying itself through the women’s demand for reparation.

Notions of identity and identification as they have been invoked in cultural studies may be useful in discussing gendered expectations, particularly when these refer to body norms. The body conveys norms of beauty and attraction/repulsion that are socially constructed and reflect aesthetic sensibilities inevitably related to gender and race power relations. The ways in which (gendered, racialized, dis/abled, etc.) bodies are portrayed (in film, literature, media, history, advertising, etc.) contribute to the ways in which individuals identify themselves. The individual constructs themselves inside discourses and representations, not outside of them. The process of self(re)construction is like that of women with FGM/C who are on a repair journey [51, 67], a journey that never really ends. The need for the patient to be continually engaged, the performativity of language through the expected discursive production of a narrative, provides both motivations and meanings for reparation that are considered essential for the process of reconstruction [51, 68]. A “successful repair” was expressed in the following way by a woman post CR “I was finally fixed! I was no longer an excised woman” [68]. The concept of identity is both strategic and positional. For excised women in France, clitoral reconstructive surgery offers the possibility of “changing sides” – they are refusing to be branded as belonging to the “barbaric” peoples; they want to get rid of the “victim” label. The question remains to determine whether the change brought through surgery is more efficient (or easier) to obtain than a change in the discourse which stigmatises FGM/C. Several authors have emphasised the need for positive, less stigmatising images in both visual and narrative terms, notably through the media and prevention campaigns carried out in countries of the South [70].

According to Butler, identifications “are never fully and finally made; they are incessantly reconstituted” [71:105] [71]. As has already been pointed out, ‘mutilated women’ is a Western concept or narrative: a way of portraying the figure of the victim and racializing the violence of the Other [72]. If this representation of FGM/C is an invention, it is nonetheless a representation within which women with FGM/C who live in the Global North construct themselves, an image to which they are (constantly) referred (by doctors, sexual partners, the media…). It is therefore an embodied representation which manifests through various social interactions and discursive practices. CR [51, 52, 66] is seeking to repair that figure, this embodied representation of the “mutilated woman,” whether she is real or imaginary.

The qualitative study carried out by O’Neill and colleagues [37] on 53 patients who had begun a CR repair journey, demonstrates this aspect through two case studies. The results show the “mutilating” effect of the discourse on those with FGM/C, that is, the performative power of language as it is used in constructing how the Other is viewed and in depicting a female identity that is lacking. Such effects do not only influence the psyche, causing mental suffering for women with FGM/C, but also provide a raft of arguments to sexual partners to “justify” the end of a relationship or claim that sexual relations are unsatisfying. In this way, if women with FGM/C go through similar experiences to other women (betrayal, separation or divorce), they tend to explain these events solely in relation the absence of their clitoris, which is an essentialisation of the very definition of what it means to be a woman, leaving the absence to cause an unbearable vulnerability.

The construction of a colonial stereotype occurs through the fetishization of the black body [73], with the aim of establishing its “difference” [74]. This fetishization of mutilated genitalia is very much in play currently, as it re-occurs within the interactions of doctors with patients, with sexual partners impregnated by discourses around FGM/C and with the women themselves, who have integrated this vision of their body and their genitalia. Fetishization has the power to reduce women solely to their alleged mutilation, with the disparaged bodily change becoming characteristic of their identity.

## Framing and naming pleasures and desires in postcolonial studies on sexualities

Questions of pleasure and desire open up a vast domain which cannot be explored through medicine alone. Sexual activity does not respond to a simple physical drive, but, like any other human activity, is embedded in social expectations and norms, very often dictated by socially constructed gender expectations. Working collaboratively with researchers in sexualities studies is highly recommended. There are several factors which should be taken into account when examining sexuality, such as notions of pleasure, desire, and consent. The disciplines of anthropology and sociology provide helpful theoretical frameworks for studying beliefs, the links between representation and practice, as well as the processes of sexual socialisation. The latter approach may be particularly fruitful, as it suggests possible intervention strategies, especially in the areas of social work and health. Examining the sexual socialisation of women who have undergone FGM/C allows us to analyse the content of the messages transmitted, the ways and styles of communication, as well as the agents and the places of socialisation [75]. Such an approach brings to light the knowledge and understanding acquired, the beliefs and the ideas received from family and friends, institutions, and the media. In studies on sexuality, it seems helpful to pay attention to the family circle and close relationships, including peers and sexual partners’ perspective and interactions, in order not to focus the attention solely on women with FGM/C and to be able to provide a more comprehensive context within which FGM/C is embedded.

Studies on sexualities should also account for a broader spectrum of sexual practices: such as homosexual relationships, masturbation, reciprocal oral sex, the significance given to these sexual moments (in terms of duration, of the sequence of events) and not focus only on penetrative (i.e., penile-vaginal) sex. The latter focus reflects a heteronormative bias and only represents *one* framework of sexuality. Furthermore, researchers should produce original studies and support their research more frequently though comparison with a control group: this could be composed of women without FGM/C after birth (for whom physiological changes induce alterations in terms of loss of pleasure or lower libido), or of young women without FGM/C who have little sexual experience (for whom knowledge of the body and of pleasure is also minimal or non-existent). Such studies might demonstrate the proximity and similarity between the two groups, rather than the difference and otherness.

Consider two studies recently carried out on women with FGM/C living in European countries. The qualitative study conducted by Ziyada and colleagues [76] took place between 2016 and 2017, among women from Sudanese and Somalian communities living in Norway. The authors show that the “cultural scenarios” [76] differed according to the ethnic group (Sudanese or Somalian), who did not share the same cultural scripts or sexual rites; the effects and the attitudes towards sex (and sexual needs) also varied according to age, sexual experience, marital status and the age on arrival in the country of immigration. The youngest participants held the views closest to the sexual norms circulating in the country of immigration and referred to more egalitarian sexual scenarios. This study shows that sexual health care needs to take these factors into account and allow for a plurality of sexual norms.

A further qualitative study was carried out by Jordal and colleagues [77] between 2016 and 2019 among 18 women from sub-Saharan origins, living in Sweden and who had undergone CR. The study, which explored the women’s level of satisfaction in relation to the CR and the changes that surgery brought, had the merit of showing that CR has the impact of reducing or eliminating feelings of stigmatisation, which is the principal result. A good proportion of women consider themselves “grateful to” doctors, which may suggest that the use of CR for black women with FGM/C living in Sweden generates a sense of ‘debt’ – reflecting the fact that they have embodied their strangeness in this country, and the doctor ‘fixed’ the problem. (One wonders whether such an attitude is similarly expressed by Swedish women who undergo breast reconstruction after a mastectomy; this comparison would be worthy of investigation).

Sexuality is embedded in power relationships, whether in the relational sphere of the couple, or between young people and their parents, or in the therapeutic relationship between doctor and patient. These interactions should be studied very carefully, in order to make the tensions present in the negotiation of sexual norms explicit and to make the agency in sexual practices visible.

## Conclusion

This paper has offered a postcolonial and feminist reading of the principal studies on CR in Europe. These studies, primarily conducted in the medical domain, show that the reasons that women give for CR reference gender identity, feelings of stigma and consequently, low self-esteem. The paper critically examines the links between representations of FGM/C and CR and the way in which these can appear as feelings of stigma, or, conversely, of gratitude. In drawing connections with other surgical procedures, the necessity to further explore the dangerous connection between an unregulated medical marketplace and neoliberal post-feminism was highlighted. Finally, the WHO is expecting a clear reference framework on CR, and more generally on surgeries affecting sexual anatomy that may or may not appropriately be described as mutilations, to which I hope this paper contributes.

The paper emphasises the importance of integrating the social sciences into studies on CR, to develop a better understanding of notions of pleasure, desire and consent considering the un/equal distribution of power and agency to act and negotiate in sexuality. Interdisciplinary studies on CR are also necessary. Comparisons with control groups (women without FGM/C; women who have experienced other type of violence, including obstetrical-gynaecological violence, etc.) could be integrated, to help avoid the construction of stigmatising “specificities” (i.e., the assumption that negative experiences or outcomes are solely due to FGM/C) and to seek to highlight plurality in sexual norms linked to sexual well-being and female sexuality. In this respect, integrating experts from the disciplines of sexualities studies, queer studies, feminist studies and postcolonial studies into ethical research committees (ERC) is strongly recommended, as currently the ERC are largely made up of experts from medical, ethical and legal disciplines.

The article critically examined the lack of knowledge and biases among health providers regarding CR and FGM/C; appropriate training for health professionals is strongly recommended. More generally, scientific advances could be better promoted, particularly through greater efforts to share scientific discoveries with young people. Multimedia supports, such as picture books, should be made available to children from a young age, promoting gender diversity images and creating the foundations for positive sexuality, which would significantly contribute to diminishing the spread of damaging beliefs and racist and sexist stereotypes.

## Data Availability

The article discusses data from published studies. The data to which the article refers are referenced in the bibliography. No original data is used in this critical article.
